# Intestinal Protective Effects of a Pomegranate Peel Extract in In Vitro and Ex Vivo Studies

**DOI:** 10.3390/ijms27031603

**Published:** 2026-02-06

**Authors:** Lucia Recinella, Alessandra Acquaviva, Annalisa Bruno, Davide Ciaramellano, Angelica Pia Centulio, Melania Dovizio, Cristina Milillo, Massimo Mozzon, Daniele Generali, Gianluca Genovesi, Giustino Orlando, Annalisa Chiavaroli, Claudio Ferrante, Patrizia Ballerini, Luigi Brunetti, Sheila Leone

**Affiliations:** 1 Department of Pharmacy, “G. d’Annunzio” University of Chieti-Pescara, 66013 Chieti, Italy; lucia.recinella@unich.it (L.R.); alessandra.acquaviva@unich.it (A.A.); d.ciaramellano@unidav.it (D.C.); angelicapia.centulio@phd.unich.it (A.P.C.); gianluca.genovesi@phd.unich.it (G.G.); giustino.orlando@unich.it (G.O.); annalisa.chiavaroli@unich.it (A.C.); claudio.ferrante@unich.it (C.F.); 2 Department of Innovative Technologies in Medicine & Dentistry, “G. d’Annunzio” University of Chieti-Pescara, 66100 Chieti, Italy; a.bruno@unich.it (A.B.); melania.dovizio@unich.it (M.D.); cristina.milillo@unich.it (C.M.); patrizia.ballerini@unich.it (P.B.); 3 Center for Advanced Studies and Technology (CAST), “G. d’Annunzio” University of Chieti-Pescara, 66100 Chieti, Italy; 4 Department of Human Sciences, Law, and Economics, Telematic University “Leonardo Da Vinci”, UNIDAV, 66100 Torrevecchia Teatina, Italy; 5 Department of Agricultural, Food and Environmental Sciences (D3A), Università Politecnica delle Marche, Via Brecce Bianche 10, 60131 Ancona, Italy; m.mozzon@univpm.it; 6 Department of Medical, Surgical and Health Sciences, University of Trieste, 34149 Trieste, Italy; dgenerali@units.it

**Keywords:** intestinal inflammation, oxidative stress, pomegranate peel

## Abstract

Recovery of nutritional and bioactive molecules by pomegranate peel (PP) has found wide applications in food and pharmaceutical industries. We investigated protective effects of a PP extract (PPE) from Mediterranean (Mazara del Vallo, Italy) on intestinal inflammation by using in vitro and ex vivo models. Reactive oxygen species (ROS) and lactate dehydrogenase (LDH) levels, as well as tight junction protein-1 (ZO-1) expression, were determined in lipopolysaccharide (LPS)-injured Caco-2 cells treated with PPE. We evaluated anti-inflammatory and antioxidant effects of PPE in isolated colon specimens of adult male mouse (C57/BL6) stimulated by LPS. Cyclooxygenase-2 (COX-2), nuclear factor-kB (NF-kB), tumor necrosis factor-α (TNF-α), interleukin-1β (IL-1β), as well as catalase (CAT), superoxide dismutase (SOD), glutathione peroxidase (GPX), and inducible nitric oxide synthase (i-NOS) gene expression was determined. We also characterized phytochemical composition of the extract through chromatographic (HPLC-UV) and spectrophotometric techniques. PPE was rich in punicalagins A and B, along with other polyphenols such as hydroxytyrosol (HT), catechin, p-coumaric acid, and rosmarinic acid. In Caco-2 cells, PPE reduced ROS generation and LDH release, restoring intestinal barrier integrity by upregulating ZO-1 expression. In addition, PPE increased SOD, CAT, and GPX and suppressed COX-2, NF-kB, TNF-α, IL-1β and i-NOS LPS-induced gene expression in colon. PPE mitigates inflammation and oxidative stress, restoring intestinal barrier function. The beneficial effects induced by the extract could be related to the pattern of polyphenolic composition, with particular regard to HT, rosmarinic acid, p-coumaric acid, catechin, as well as punicalagins A and B.

## 1. Introduction

In the last decade, the focus on sustainability has led to an increased interest in utilizing agro-industrial by-products, driven by the growing awareness of environmental impact and the potential of waste materials [1]. In particular, the recovery of nutritional and bioactive molecules by pomegranate (*Punica granatum*) peel has found wide applications in food and pharmaceutical industries [2]. Pomegranate belongs to the Lythraceae family and is highly valued for its numerous health benefits [3]. Preclinical and clinical studies demonstrated antidiabetic, antiviral [4], as well as anticancer properties [5,6] of pomegranate. Additionally, pomegranate exhibited anti-inflammatory and antioxidant activities by neutralizing reactive oxygen species (ROS) and inhibiting transcription factors like nuclear factor-kB (NF-kB) [7]. Different studies demonstrated therapeutic effects of pomegranate fruit and flowers, as well as their ellagitannins, against gastrointestinal diseases, including ulcerative colitis. In this context, compounds like punicalagin and ellagic acid, which are found in pomegranate, can inhibit oxidative and inflammatory processes, thus regulating the intestinal barrier function [8]. Similarly, pomegranate peel (PP) has been shown to improve gut microbiota and alleviate chronic intestinal inflammation thanks to the presence of punicalagin, ellagic acid, and other active ingredients [9,10,11,12]. Ellagitannins were identified as key players in inhibiting primary intestinal inflammation by regulating key proteins, such as NF-kB, MAPK, p70S6K, and STAT3, involved in various inflammatory pathways. Moreover, they can also reduce oxidative stress, relieving the symptoms of ulcerative colitis [7,8].

On the basis of these findings, in this study we aimed to evaluate the potential protective effects of a pomegranate peel extract (PPE) from Mediterranean (Mazara del Vallo, Italy) waste material obtained during pomegranate juice production. We determined ROS and lactate dehydrogenase (LDH) levels, as well as tight junction protein-1 (ZO-1) expression in lipopolysaccharide (LPS)-injured Caco-2 cells treated with PPE at concentrations of 1, 10, and 100 µg/mL. In addition, we evaluated the potential anti-inflammatory and antioxidant effects of the extract in an ex vivo model of colitis constituted by isolated mouse colon specimens stimulated by LPS. This involved evaluation of cyclooxygenase-2 (COX-2), NF-kB, tumor necrosis factor-α (TNF-α), interleukin-1β (IL-1β), as well as catalase (CAT), superoxide dismutase (SOD), glutathione peroxidase (GPX), and inducible nitric oxide synthase (i-NOS) gene expression by RT-PCR. We also characterized the phytochemical composition of the extract through chromatographic (HPLC-UV) and spectrophotometric techniques.

## 2. Results and Discussion

### 2.1. HPLC-UV Analysis

**Figure 1:**
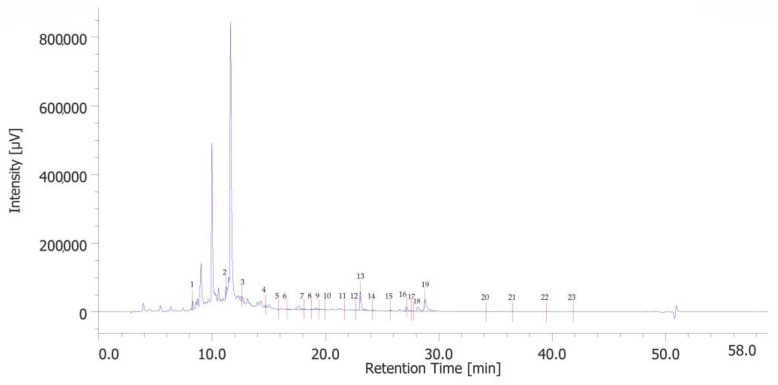
HPLC-UV chromatogram of identified phenolic compounds of PPE. The prominent compounds are 3-hydroxytyrosol (peak #2), catechin (peak #4), p-coumaric acid (peak #13), and rosmarinic acid (peak #19).

The chromatographic analysis identified 23 phenolic compounds. 3-hydroxytyrosol (peak #2), catechin (peak #4), p-coumaric acid (peak #13), and rosmarinic acid (peak #19) were the most abundant phenols (Figure 1). Quantitative determination is reported in Table S2 in the Supplementary Materials.

The analysis of the extract shows some consistency with the findings from previous studies [13]. Specifically, phenolic acids are predominant among the various phenolic subclasses, which may be attributed to the extraction methods and the specific conditions under which the pomegranates were cultivated [14].

### 2.2. Quantification of Ellagitannins

**Figure 2:**
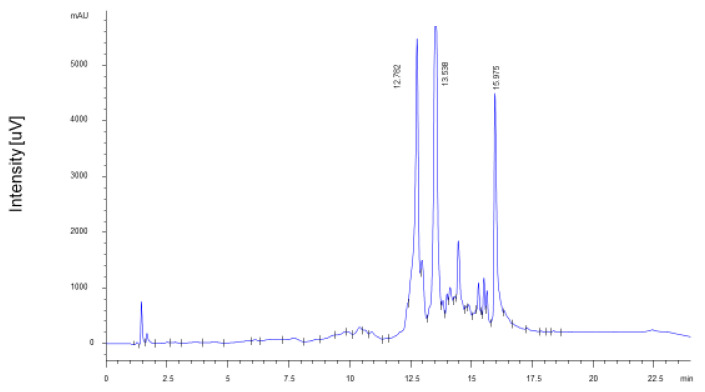
HPLC-UV chromatogram of ellagitannins of PPE. Identified peaks are: punicalagin A (Rt = 12.782 min), punicalagin B (Rt = 13.438 min), ellagic acid (Rt = 15.975 min).

The extract of fresh PP is characterized by the almost exclusive presence of punicalagin stereoisomers in similar proportions (A = 43%; B = 57%), as previously described in the literature [15,16,17]. The total amount of punicalagins was 14.14 ± 0.71 g/100 g of dry peel (6.09 ± 0.31 g/100 g punicalagin A; 8.05 ± 0.40 g/100 g punicalagin B) (Figure 2), which corresponds to 56% of the identified ellagitannins. The total PPE yielded 34.15 ± 1.63 g/100 g of dry peel. Literature data indicate that punicalagins are the most important bioactive components in vivo and are characterized by high absorption rate and bioavailability [18]. Among the degradation products of punicalagins, ellagic acid accounted for 11.13 ± 0.56 g/100 g of dry peel, while punicalins A and B were not detected.

### 2.3. In Vitro Studies

In the first experimental phase, we investigated the potential protective effects of the extract (PPE, 1, 10, 100 μg/mL) on oxidative stress and ROS generation utilizing a model of human colorectal adenocarcinoma constituted by Caco-2 cells treated with LPS. This model has proven to be useful for evaluating the protective effects of herbal extracts on intestinal epithelium function, since LPS is capable not only of inducing oxidative stress but also increasing membrane permeability and disrupting tight junctions of intestinal epithelial cells [19].

**Figure 3:**
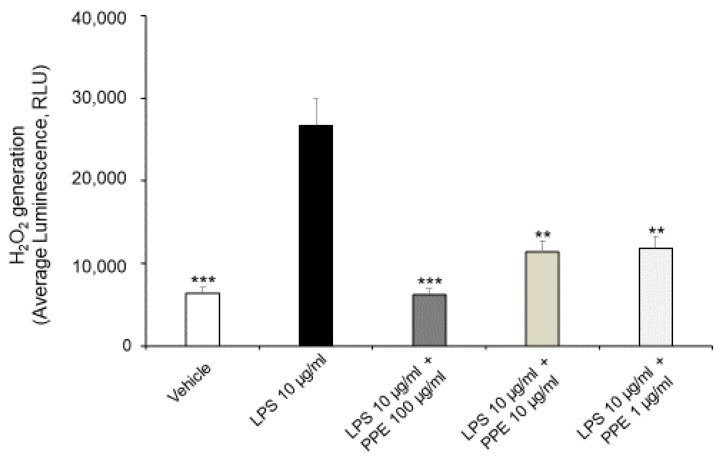
Effects of PPE on ROS generation in LPS-injured Caco-2 cells. The graph shows ROS generation in LPS-injured Caco-2 cells (Average luminescence, RLU) treated with PPE (1, 10, 100 μg/mL). Each value represents the mean ± SEM of at least three independent experiments. ** *p* < 0.01 and *** *p* < 0.001 vs LPS (N = 3–4, one-way ANOVA followed by Bonferroni post-test).

As shown in Figure 3, the treatment of Caco-2 cells with LPS (10 μg/mL) for 24 h promotes ROS generation, with a statistically significant increase in H_2_O_2_ generation. In this context, the co-treatment with PPE (1, 10, 100 μg/mL) prevented this effect at all tested concentrations, as shown by H_2_O_2_ generation comparable to the control condition.

Our present findings are in agreement with those of Rak-Pasikowska and collaborators (2024) [20], which showed decreased ROS/RNS levels following PPE administration in the spleen of rats with metabolic syndrome. Moreover, pomegranate peel polyphenols, as well as punicalagins and ellagic acid, were also found effective in decreasing ROS production in RAW264.7 macrophages challenged with LPS [21], thus confirming their potential antioxidant effects.

**Figure 4:**
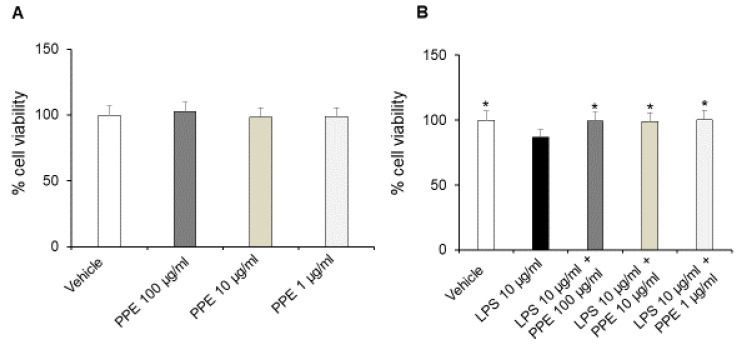
Effects of PPE on Caco-2 cells viability in basal conditions and after LPS exposure. The graph shows cell viability (%) in basal condition (panel (**A**)) and after LPS-injured (panel (**B**)), in Caco-2 cells co-treated with PPE (1–10–100 μg/mL). Each value represents the mean ± SEM of at least three independent experiments. (**A**) N = 4–6, (**B**) * *p* < 0.05 vs LPS (N = 5–6, one-way ANOVA followed by Bonferroni post-test).

The treatment of Caco-2 cells with increasing concentrations of PPE (1, 10, 100 μg/mL) up to 24 h did not affect the viability and metabolic activity of cells in basal conditions (Figure 4A).

On the other hand, incubation with LPS (10 μg/mL) for 24 h significantly reduced the cell viability of Caco-2 cells. The co-treatment with the extract (1, 10, 100 μg/mL) prevented this effect at all tested concentrations, as shown by cell viability percentages comparable to the control condition (Figure 4B). In this context, the protective effects induced by PPE were also recently confirmed by Alami and collaborators (2024) [22]. In addition, punicalagins were found to inhibit LPS-mediated cytotoxicity in BV2 microglial cells, further confirming the protective activities induced by PPE [23].

**Figure 5:**
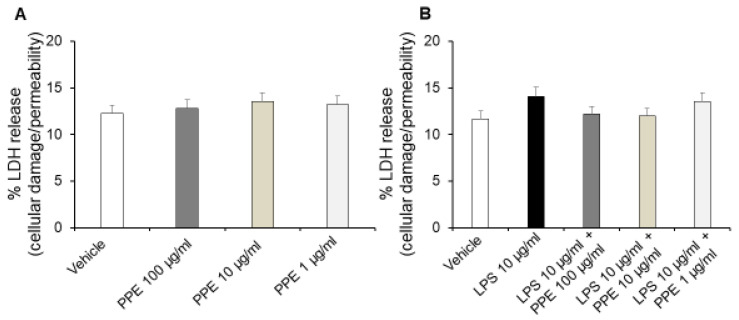
Effects of PPE on cellular damage and permeability in basal conditions and after LPS exposure. The graph shows LDH release (%) in basal condition (panel (**A**)) and after LPS-injured in Caco-2 cells co-treated with PPE (1–10–100 μg/mL) (panel (**B**)). Each value represents the mean ± SEM of at least three independent experiments. (**A**) N = 4, (**B**) (N = 3, Student’s *t*-test).

Interestingly, treatment of Caco-2 cells with increasing concentrations of PPE (1, 10, 100 μg/mL) for up to 24 h did not affect release of LDH (Figure 5A), a stable cytoplasmic enzyme that is found in all cells. LDH is rapidly released into the cell culture supernatant, and the assessment of its metabolic activity represents a reliable indicator of cell membrane damage and cell death directly resulting from increased cell permeability [24].

By contrast, treatment of Caco-2 cells with LPS (10 μg/mL) for 24 h significantly increases the extracellular release of LDH. Additionally, co-treatment with PPE (1, 10, 100 μg/mL) prevented this effect at all tested concentrations, as indicated by LDH release comparable to the control condition (Figure 5B).

In agreement, previous studies reported that HT can reduce cell death and inhibit LDH release in a concentration-dependent manner in normal rat brain slices [25]. Moreover, the inhibitory effects of catechin, rosmarinic acid and p-coumaric acid on LDH activity are also well known, thus suggesting their involvement in protective effects induced by PPE [26,27,28].

Finally, we evaluated the capacity of the extract to up-regulate expression of ZO-1, a peripheral membrane protein specifically associated with the cytoplasmic surface of tight junctions [29].

**Figure 6:**
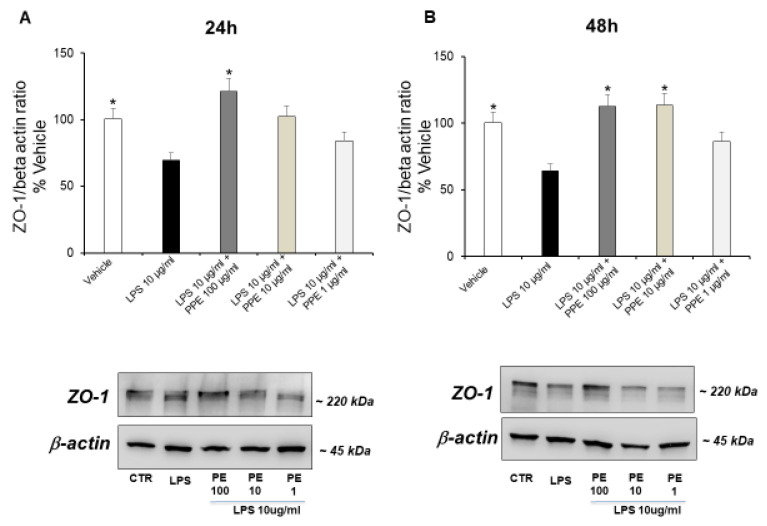
Effects of PPE on tight junction protein expression (ZO-1) in LPS-injured Caco-2 cells. The results are expressed as the protein expression level (normalized to β-actin) relative to vehicle. Each value represents the mean ± SEM of at least three independent experiments. (**A**) * *p* < 0.05 vs. LPS (N = 3, Student’s *t*-test) (**B**) * *p* < 0.05 vs. LPS (N = 5–7, Student’s *t*-test).

The treatment of Caco-2 cells with LPS (10 μg/mL) up to 24 (Figure 6A) and 48 h (Figure 6B) significantly down-regulated the ZO-1 expression, in a time-dependent manner. This data supports the damage to membrane integrity and the altered cell permeability caused by LPS.

The co-treatment with PPE at a dose of 100 μg/mL (24 h, Figure 6A) and PPE at a dose of 10 and 100 μg/mL (48 h, Figure 6B) prevented this effect, as indicated by the ZO-1 expression levels comparable to the control condition. Quantitative determination is reported in the Supplementary Materials. In agreement, the significant role of PPE in preserving the integrity of the intestinal barrier was also recently suggested by Zhang and collaborators (2024) [30]. In this context, PPE treatment was found able to increase the expression of key proteins involved in tight junction formation, including ZO-1, in colon tissues of diarrheal irritable bowel syndrome rats compared to control group [30].

HT was found to have protective activities on the ZO-1 and the integrity of the intestinal barrier. It enhances ZO-1 expression and levels, particularly in cases of gut inflammation or other harmful conditions. This action helps to restore the structure and functionality of the gut barrier, reducing inflammation and oxidative stress by lowering paracellular permeability and promoting tighter junctions between cells [31]. Moreover, rosmarinic acid was also found to restore intestinal barrier integrity through upregulation of key tight junction proteins, including ZO-1 in colon [32].

We can hypothesize that the protective activities of PPE could be related to its phytochemical composition, with particular regard to HT, catechin, rosmarinic acid, and p-coumaric acid.

### 2.4. Ex Vivo Studies

In the second experimental step, we evaluated the potential beneficial activities induced by the extract (PPE, 1, 10, 100 μg/mL) in isolated mouse colon specimens stimulated with LPS, which represents a validated model of colitis [33,34].

In colonic inflammation, NF-kB translocates into the nucleus, activating the transcription of different pro-inflammatory cytokines, including TNF-α and IL-1β. These cytokines are responsible for the tissue damage, the recruitment of inflammatory cells, as well as creating a positive feedback loop with NF-kB, which amplifies even more the inflammatory response [35].

**Figure 7:**
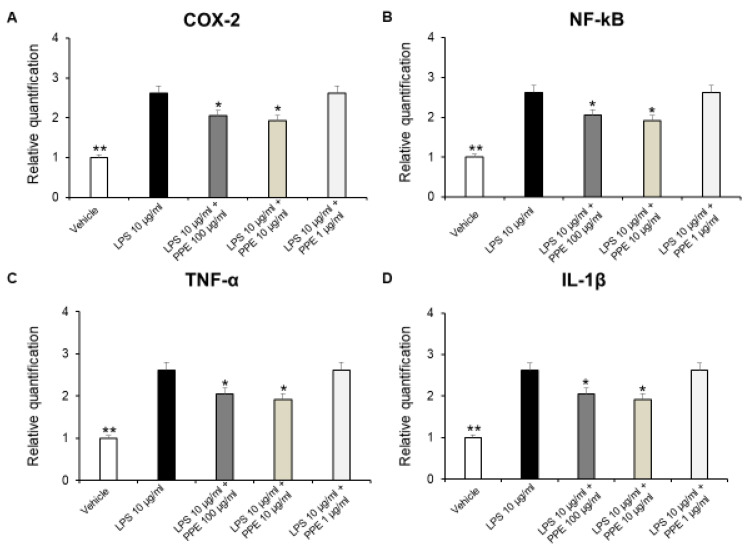
Real-time PCR of COX-2, NF-kB, TNF-α, and IL-1β. Effects of PPE (1, 10, 100 µg/mL) on LPS-induced gene expression of COX-2 (**A**), NF-kB (**B**), TNF-α (**C**), and IL-1β (**D**) in mice colon specimens. * *p* < 0.05, ** *p* < 0.005 vs. LPS. Each value represents the mean ± SEM of at least three independent experiments.

In addition, a large body of evidence suggests that COX-2 expression is induced in the large intestinal epithelium following colon inflammation [36,37]. Therefore, we investigated the effects of PPE on gene expression of pro-inflammatory mediators, such as COX-2, NF-kB, TNF-α, and IL-1β on isolated LPS-stimulated colon specimens, by RT-PCR analysis. In our ex vivo model, we showed that PPE (10–100 μg/mL) was able to reduce COX-2, NF-kB, TNF-α, and IL-1β gene expression in LPS-injured colon tissue (Figure 7A–D).

These results agreed with the anti-inflammatory effects induced by a pomegranate peel water extract tested both in vitro in human intestinal Caco-2 cells and ex vivo in porcine colonic tissue explants [38]. In agreement, the anti-inflammatory effects induced by HT are also well known. HT administration was effective in reducing pro-inflammatory cytokines, such as IL-6, IL-1β, or TNF-α in dextrane sulfate sodium (DSS)-induced colitis, in mice [39,40]. In addition, catechins were found able to reduce expression of TNF-α and IL-6 in a mouse model of DSS-induced colitis [41]. The protective effects against DSS-induced colitis were also reported for rosmarinic acid [42], p-coumaric acid [43], and punicalagins [44]. Colon inflammation is also characterized by an imbalance between the production of ROS and the body’s antioxidant defenses. This state, known as oxidative stress, is a key driver of tissue damage and perpetuates the inflammatory response.

The enzymes CAT, GPX, and SOD are crucial components of the antioxidant defense system, while i-NOS contributes to the production of ROS, which also cause damage [45].

**Figure 8:**
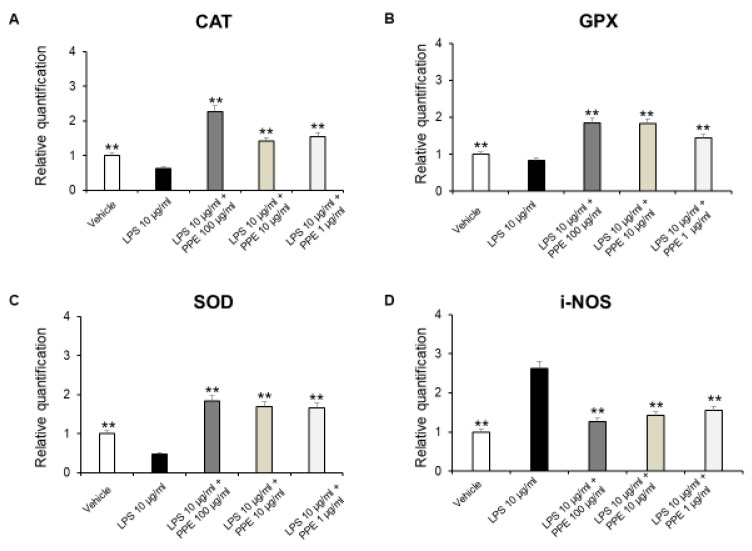
Real-time PCR of CAT, GPX, SOD, and i-NOS. Effects of PPE (1–10-100 µg/mL) on LPS-induced catalase (CAT) (**A**), glutathione peroxidase (GPX) (**B**), superoxide dismutase (SOD) (**C**), and inducible nitric oxide synthase (i-NOS) (**D**) gene expression in mice colon specimens. ** *p* < 0.005 vs. LPS. Each value represents the mean ± SEM of at least three independent experiments. (**A**) N = 4–6, (**B**) (N = 5–6, one-way ANOVA followed by Bonferroni post-test).

As shown in Figure 8A–D, PPE was able to induce antioxidant effects by increasing antioxidant enzymes, including SOD, CAT, and GPX, as well as decreasing i-NOS gene expression.

Accordingly, besides the well-known effects of punicalagins in the inflammatory process, rosmarinic acid was also effective in suppressing i-NOS gene expression, in DSS-induced colitis [46]. Furthermore, p-coumaric acid was also able to increase SOD, CAT, and GPx levels in colon carcinogenesis in a dose-dependent manner [47]. Catechins were found to be able to reverse histological lesions and increase the expression of antioxidant markers, including SOD, GPx, and CAT, in a mouse model of DSS-induced colitis [41]. Finally, HT increased activities of SOD and CAT, thus restoring the pro-oxidant/antioxidant balance, in colitis [48].

## 3. Materials and Methods

### 3.1. Extract Preparation

Pomegranate peels of the Wonderful variety were supplied by Naturalia Ingredients Srl (Mazara del Vallo, TP, Italy). The fresh fruits were peeled by hand. The peels were crushed, freeze-dried, and ground. Lyophilization was selected as the drying method to prevent the thermal and oxidative degradation of sensitive phenolic compounds, particularly ellagitannins, which are highly susceptible to damage during conventional thermal drying [49]. This process ensured that the chemical profile of the starting material remained as close as possible to the fresh state. The polyphenolic substances were extracted by shaking the pomegranate peel powder in 25% ethanol at 45 °C for 35 min. This specific hydroalcoholic concentration was chosen following the trend observed in the literature, where lower ethanol percentages (e.g., 25–30%) demonstrate higher efficiency for pomegranate peel compared to higher concentrations [50], while further enhancing the ‘green’ profile of the process. Extraction parameters (45 °C, 35 min) were selected to balance recovery efficiency and compound stability, as moderate temperatures prevent the thermal degradation of thermolabile punicalagins [51]. No pH adjustment was performed to preserve the natural acidity of the matrix—which favors phenolic stability—and to mimic the conditions of a domestic or ‘green’ food industry preparation [17,52].

The solid/liquid ratio was 1:5 (*w*/*v*). The liquid phase was recovered by centrifugation and freeze-dried. The resulting extract was stored under vacuum at −20 °C until use. These storage conditions are widely recognized to preserve the antioxidant capacity and chemical integrity of pomegranate polyphenols, particularly ellagitannins, which maintain high stability at sub-zero temperatures [17].

### 3.2. HPLC-UV Analysis

The lyophilized extract was reconstituted in HPLC-grade water (the same vehicle used for biological assays) by weighing 80 mg of lyophilized sample using a Precisa XT220A balance (Micro Precision Calibration Inc., Grass Valley, CA, USA), homogenized together with 2 mL of distilled water. The lyophilized powder dissolved completely in water. This choice was made to maintain compatibility with the aqueous HPLC mobile phase, preventing chromatographic artifacts, and to align with green chemistry principles by avoiding unnecessary organic solvents. Furthermore, water-based reconstitution reflects the solubility profile expected in domestic or food-grade applications. Ultrasound-assisted extraction (UAE) of the homogenate was performed at 60 °C for 20 min, maximum power. The sample was then centrifuged, filtered with PTFE 0.22 μm, and further diluted to 5 mg/mL before injection into HPLC.

The HPLC apparatus consisted of two chromatographic pumps (PU-2080 PLUS), degasser (DG-2080-54-line), mixer (2080-32), UV detector, autosampler (AS-2057 PLUS), and column thermostat (CO-2060 PLUS), all from Jasco, Tokyo, Japan. Integration was performed using ChromNAV2 Chromatography software (version 2.2.2.3). Standards were purchased from Sigma-Aldrich (Milan, Italy). The separation was conducted using an Infinity lab Poroshell 120-SB reverse phase column (C18, 150 × 4.6 mm i.d., 2.7 µm; Agilent, Santa Clara, CA, USA) at a flow rate of 0.6 mL/min. The column’s temperature was maintained at 30 °C. The chromatographic run time was 60 min, starting from the following separation conditions: 97% water added with 0.1% formic acid, and 3% methanol added with 0.1% formic acid, in gradient elution mode. Gradient details are listed in Table S1 in Supplementary Materials. The quantitative analysis of phenolic compounds was carried out using a UV detector set to 254 nm. This wavelength was selected as a strategic ‘compromise’ for a comprehensive multi-target screening, allowing the simultaneous detection of 38 different compounds in a single run [53]. To ensure analytical consistency, all samples were analyzed in duplicate using a high-precision automatic injector. The volume of injection was 5 µL. Quantification was done through 7-point calibration curves, with linearity coefficients (R2) > 0.999, in the concentration range 2–140 µg/mL, as previously described [54].

#### Determination of Ellagitannins

The extract was analyzed for its bioactive ellagitannin content by HPLC-UV in an Agilent 1100 system equipped with an online degasser, a quaternary pump, a Zorbax C18 Eclipse Plus column (3.0 × 150 mm, 3.5 µm), and a variable wavelength detector at 260 nm. The solvents were 0.15% phosphoric acid in water (A) and 0.15% phosphoric acid in acetonitrile. The mobile phase flowed at 0.7 mL/min with the following elution program: 0 min, 99% A; 1.5 min 99% A; 3.0 min, 95.5% A; 5 min, 95.5% A; 8.5 min, 93% A; 13.8 min, 75% A; 14.4 min, 10% A; and 20 min, 10% A.

Pure analytical standards were provided by Sigma-Aldrich (Saint Louis, MO, USA) (ellagic acid) and PhytoLab GmbH & Co KG (Vestenbergsgreuth, Germany) (punicalagin A + B mixture and punicalin A + B mixture). The standard solutions for the calibration curve were prepared in methanol/water 1:1 (*v*/*v*) in the range of 0.1–1 mg/mL. The injection volume for standards and samples was 5 µL.

### 3.3. Cell Culture

The human colorectal adenocarcinoma cell line Caco-2 was purchased from ATCC (Manassas, VA, USA) and cultured in Dulbecco’s Modified Eagle’s Medium supplemented with 10% (*v*/*v*) heat-inactivated fetal bovine serum (FBS), 2 mM L-glutamine, 100 μg/mL penicillin, and 100 μg/mL streptomycin, according to the manufacturer’s instructions. LPS and all the components used for cell cultures were purchased from Sigma-Aldrich (Milan, Italy). The extract was dissolved in distilled water to avoid ethanol-induced cytotoxicity.

#### 3.3.1. ROS Generation

The ROS production was evaluated using the ROS-GloTM H2O2 Assay kit (Promega Corporation, Madison, WI, USA) following the manufacturer’s recommendations and as previously described [55,56]. Briefly, 1.5 × 10^4^ cells/well were seeded in white opaque 96-well plates (Corning, Sigma-Aldrich) and left to adhere overnight. Then, cells were treated with LPS (Sigma-Aldrich, St. Louis, MO, USA) (10 μg/mL) and increasing concentrations of PPE (1–10–100 μg/mL) for up to 24 h. The H_2_O_2_ substrate solution was added to the cells after 18 h of treatment, and cells were incubated for a further 6 h in a humidified atmosphere of 5% CO_2_ at 37 °C. At the end of the treatment, 100 μL of ROS-Glo detection solution was added to each well, incubated at room temperature for 20 min, and the luminescence was measured with a SynergyH1 BioTek spectrophotometer (Agilent, Santa Clara, CA, USA). To account for background luminescence, values from wells containing only medium or medium with PPE (at each concentration) were subtracted from the corresponding experimental wells.

#### 3.3.2. Cell Viability

Cell viability was assessed using the CyQUANT™ XTT Cell Viability Assay (Thermo Fisher Scientific, Waltham, MA, USA) following the manufacturer’s protocol. Caco-2 cells (1.5 × 10^4^ cells/well) were seeded into 96-multiwell and treated with LPS (10 μg/mL) and/or increasing concentrations of PPE (1–10–100 μg/mL) for up to 24 h. In the last 4 h of treatment, the XTT reagent was added to each well and incubated at 37 °C. Absorbance was measured at 450 nm with a reference wavelength of 660 nm using a SynergyH1 BioTek spectrophotometer (Agilent, Santa Clara, CA, USA). To account for background absorbance, values from wells containing only medium or medium with PPE (at each concentration) were subtracted from the corresponding experimental wells.

#### 3.3.3. Lactate Dehydrogenase (LDH) Assay

The released LDH in culture supernatants was evaluated using the CytoTox 96^®^ Non-Radioactive Cytotoxicity Assay (Promega Corporation, Milan, Italy) following the manufacturer’s protocol. Briefly, Caco-2 cells (1.5 × 10^4^ cells/well) were seeded into 96-well plates and left to adhere overnight. Cells were treated with LPS (10 μg/mL) and/or increasing concentrations of PPE (1–10–100 μg/mL) for up to 24 h. Following treatment, the culture medium was collected and incubated with CytoTox 96^®^ reagent. LDH release into the medium was quantified by measuring absorbance at 490 nm using a SynergyH1 BioTek spectrophotometer (Agilent, Santa Clara, CA, USA). To correct for background absorbance, values from wells containing only medium or medium with PPE (at each concentration) were subtracted from the corresponding experimental wells. Results were expressed as a percentage of maximum LDH release assessed in control wells in which cells were lysed to obtain the maximum release of LDH in our experimental conditions.

### 3.4. Western Blot

Caco-2 cells (1 × 10^6^ cells/well) were seeded into 6-well plates and left to adhere overnight. Cells were treated with LPS (10 μg/mL) and/or increasing concentrations of PPE (1, 10, 100 μg/mL) for 24 and 48 h. Protein expression of ZO-1 was assessed by Western blot, as previously described [57]. Briefly, cells were lysed, and protein concentrations were determined using the Bradford assay. Equal amounts of protein (30 µg) were loaded and separated by SDS-PAGE, followed by transfer to PVDF membranes. The membranes were blocked for 1 h in 5% non-fat milk in PBS-Tween20 (0.1%), then incubated overnight at 4 °C with a primary antibody against ZO-1 (1:1000, Cell Signaling Technology, Danvers, MA, USA). After washing, membranes were incubated with a secondary antibody (1:5000, Bio-Rad, Milan, Italy) for 1 h at room temperature.

Membranes were developed using ECL Western blotting detection reagents (Bio-Rad) and analyzed using software Alliance 1D MAX associated with the Alliance Mini (UVITEC, Cambridge, UK) and ImageJ software version 1.54g. Band intensity was quantified and normalized to the expression of β-actin to ensure equal protein loading across samples.

### 3.5. Ex Vivo Studies

Adult C57/BL6 male mice (3-month-old, weight 20–25 g) (n = 30, 6 for each experimental group) were housed in Plexiglas cages.

After collection, isolated mouse colon specimens were maintained in a humidified incubator with 5% CO_2_ at 37 °C for 4 h, in RPMI buffer with added bacterial LPS (Sigma-Aldrich, St. Louis, MO, USA) (10 μg/mL) (incubation period) [33,34,58]. The extract was dissolved in distilled water to avoid ethanol-induced cytotoxicity. During the incubation period, colon specimens were treated with PPE (1, 10, 100 µg/mL) or vehicle. After collection, total RNA was extracted from the colon specimens using TRI Reagent (Sigma-Aldrich, St. Louis, MO, USA), according to the manufacturer’s protocol. Contaminating DNA was removed using 2 units of RNase-free DNase 1 (DNA-free kit, Ambion, Austin, TX, USA). The RNA concentration was quantified at 260 nm by spectrophotometer reading (BioPhotometer, Eppendorf, Hamburg, Germany), and its purity was assessed by the ratio at 260 and 280 nm readings. The quality of the extracted RNA samples was also determined by electrophoresis through agarose gels and staining with ethidium bromide, under UV light. One microgram of total RNA extracted from each sample in a 20 µL reaction volume was reverse transcribed using High-Capacity cDNA Reverse Transcription Kit (Thermo Fisher Scientific Inc., Monza, Italy). Reactions were incubated in a 2720 Thermal Cycler (Thermo Fisher Scientific Inc., Monza, Italy) initially at 25 °C for 10 min, then at 37 °C for 120 min, and finally at 85 °C for 5 s. Gene expression of COX-2, TNF-α, NF-kB, IL-1β, CAT, SOD, GPX, and i-NOS was determined by quantitative real-time PCR using TaqMan probe-based chemistry, as previously described [34]. β-actin (Thermo Fisher Scientific Inc., Monza, Italy, Part No. 4352340E) was used as the housekeeping gene. The real-time PCR was carried out in triplicate for each cDNA sample in relation to each of the investigated genes. Data were elaborated with Sequence Detection System (SDS) software version 2.3 (Thermo Fisher Scientific Inc., Monza, Italy). Gene expression was relatively quantified by the comparative 2^−∆∆Ct^ method [59]. In regard to gene expression analysis, we confirm the stability of β-actin gene expression analysis under the evaluated experimental conditions.

### 3.6. Statistical Analysis

Data were analyzed by licensed software GraphPad Prism version 6.0 (Graphpad Software Inc., San Diego, CA, USA). Analysis of means ± SEM for each experimental group was performed by *t*-test and one-way analysis of variance (ANOVA), followed by the Bonferroni post hoc test.

The number of animals randomized for each experimental group was calculated on the basis of the “Resource Equation” N = (E + T)/T (10 ≤ E ≤ 20) [58].

## 4. Conclusions

In conclusion, our study provides compelling evidence that PPE could represent a potential adjuvant strategy to defuse colitis-associated inflammatory process. PPE could also exert a multifaceted protective effect by fundamentally reinforcing the intestinal mucosal barrier. By restoring the expression of ZO-1 and stabilizing the structural integrity of tight junctions, PPE preserves the selective permeability of the epithelium, known as a critical factor in preventing the systemic translocation of luminal toxins and pathogens that drive chronic gut inflammation. The protective effects exerted by PPE in colon could be linked to its content in polyphenols, with particular regard to HT, rosmarinic acid, p-coumaric acid, catechin, as well as punicalagins A and B. Thereafter, PPE could offer a promising alternative to conventional therapeutic approaches, which often have limitations like side effects or limited efficacy. This study provides a potential mechanistic foundation that justifies further clinical investigation into PPE as a functional tool for maintaining long-term intestinal health and mucosal integrity.

## Data Availability

The data that support the findings of this study are available from the corresponding author upon reasonable request.

## Supplementary Materials

The following supporting information can be downloaded at: https://www.mdpi.com/article/10.3390/ijms27031603/s1.

## Abbreviations

The following abbreviations are used in this manuscript:

ROS	reactive oxygen species
LPS	lipopolysaccharide
GPX	glutathione peroxidase
CAT	catalase
i-NOS	inducible nitric oxide synthase
COX-2	cyclooxygenase
TNF-α	tumor necrosis factor- α
NF-kB	nuclear factor-kB
IL	interleukin
LDH	lactate dehydrogenase
PPE	pomegranate peel extract
ZO-1	tight junction protein (ZO-1)
