# Supplementary material for: Intestinal Protective Effects of a Pomegranate Peel Extract in In Vitro and Ex Vivo Studies
Source: Int J Mol Sci. 2026 Feb 6;27(3):1603. doi: 10.3390/ijms27031603 (PMC12898482; doi:10.3390/ijms27031603)
Supplement: Supplementary file 1 [file ijms-27-01603-s001.zip › ijms-4082143-supplementary.pdf]

Supplementary Materials for:

Intestinal protective effects of a pomegranate peel extract in *in vitro* and *ex-vivo* studies

Lucia Recinella [1,#], Alessandra Acquaviva [1], Annalisa Bruno [2,3#], Davide Ciaramellano [1,4], Angelica Pia Centulio [1], Melania Dovizio [2,3], Cristina Milillo [2], Massimo Mozzon [5], Daniele Generali [6], Gianluca Genovesi [1], Giustino Orlando [1], Annalisa Chiavaroli [1], Claudio Ferrante [1], Patrizia Ballerini [2,3], Luigi Brunetti [1,*], Sheila Leone [1]

[1] Department of Pharmacy, G. d'Annunzio University of Chieti-Pescara, 66013, Chieti, Italy; lucia.recinella@unich.it (L.R.); alessandra.acquaviva@unich.it (A.A.); d.ciaramellano@unidav.it (D.C.); angelicapia.centulio@phd.unich.it (A.P.C.); gianluca.genovesi@phd.unich.it (G.G.); giustino.orlando@unich.it (G.O.); annalisa.chiavaroli@unich.it (A.C.); claudio.ferrante@unich.it (C.F.); luigi.brunetti@unich.it (L.B.); sheila.leone@unich.it (S.L.).
[2] Department of Innovative Technologies in Medicine & Dentistry, "G. d'Annunzio" University of Chieti-Pescara, 66100 Chieti, Italy; a.bruno@unich.it (A.B.); melania.dovizio@unich.it (M.D.); cristina.milillo@unich.it (C.M.); patrizia.ballerini@unich.it (P.B.).
[3] Center for Advanced Studies and Technology (CAST), "G. d'Annunzio" University of Chieti-Pescara, 66100 Chieti, Italy; a.bruno@unich.it (A.B.); melania.dovizio@unich.it (M.D.); patrizia.ballerini@unich.it (P.B.);
[4] Department of Human Sciences, Law, and Economics, Telematic University "Leonardo Da Vinci", UNIDAV, 66100, Torrevecchia Teatina, Italy; d.ciaramellano@unidav.it (D.C.).
[5] Department of Agricultural, Food and Environmental Sciences (D3A), Università Politecnica delle Marche, Via Brecce Bianche 10, 60131 Ancona, Italy; m.mozzon@univpm.it (M.M.).
[6] Department of Medical, Surgical and Health Sciences, University of Trieste, 34149 Trieste, Italy; dgenerali@units.it (D.G.).
* Correspondence: luigi.brunetti@unich.it; Tel.: +39-0871-3554758
[#] These authors equally contributed to the manuscript.

1. Materials and Methods

1.1. HPLC-UV analysis

The lyophilized extract was reconstituted in HPLC-grade water (the same vehicle used for biological assays) by weighing 80 mg of lyophilized sample using a Precisa XT220A balance (Micro Precision Calibration Inc., Grass Valley, CA, USA), homogenized together with 2 ml of distilled water. The lyophilized powder dissolved completely in water. This choice was made to maintain compatibility with the aqueous HPLC mobile phase, preventing chromatographic artifacts, and to align with green chemistry principles by avoiding unnecessary organic solvents. Furthermore, water-based reconstitution reflects the solubility profile expected in domestic or food-grade applications. Ultrasound-assisted extraction (UAE) of the homogenate was performed at 60°C for 20 min, maximum power. The sample was then centrifuged, filtered with PTFE 0.22 µm, and further diluted to 5 mg/ml before injection into HPLC.

The HPLC apparatus consisted of two chromatographic pumps (PU-2080 PLUS), degasser (DG-2080-54-line), mixer (2080-32), UV detector, autosampler (AS-2057 PLUS) and column thermostat (CO-2060 PLUS), all from Jasco, Tokyo, Japan. Integration was performed using ChromNAV2 Chromatography software. Standards were purchased from Sigma-Aldrich (Milan, Italy). The separation was conducted using an Infinity lab Poroshell 120-SB reverse phase column (C18, 150 × 4.6 mm i.d., 2.7 µm; Agilent, Santa Clara, CA, USA) at a flow rate of 0.6 ml/min. The column's temperature was maintained at 30°C. The chromatographic run time was 60 min, starting from the following separation conditions: 97 % water added with 0.1 % formic acid, and 3 % methanol added with 0.1 % formic acid, in gradient elution mode. Gradient details are listed in Table S1 in supplementary materials. The quantitative analysis of phenolic compounds was carried out using a UV detector set to 254 nm. This wavelength was selected as a strategic 'compromise' for a comprehensive multi-target screening, allowing the simultaneous detection of 38 different compounds in a single run [1]. To ensure

analytical consistency, all samples were analyzed in duplicate using a high-precision automatic injector. The volume of injection was 5 μL. Quantification was done through 7-point calibration curves, with linearity coefficients (R2) > 0.999, in the concentration range 2–140 μg/ml, as previously described [2].

Table S1. Gradient details of HPLC-UV analysis for phenolic compounds determination.

TIME (min)	COMPOSITION A% (water + formic acid 0.1%)	COMPOSITION B% (methanol + formic acid 0.1%)	FLOW (ml/min)
1	97	3	0.6
5	77	23	0.6
12	73	27	0.6
18	57	43	0.6
25	52	48	0.6
32	50	50	0.6
34	50	50	0.6
37	35	65	0.6
38	25	75	0.6
40	25	75	0.6
41	5	95	0.6
47	5	95	0.6
48	97	3	0.6

Table S2. Retention times (Rt, min) and quantities (μg/ml) of the identified phenolic compounds in the PPE (n.q.= not quantified). Data are expressed as means of two replicates ± SD.

#	Analytes	Rt (min)	Quantity (μg/ml)		
1	Gallic acid	8.80	9.927	±	0.530
2	3-Hydroxytyrosol	11.71	103.102	±	0.612
3	Caftaric acid	12.93	13.179	±	0.526
4	Catechin	14.80	41.274	±	1.284
5	4-Hydroxybenzoic acid	16.20	n.q.		
6	Loganic acid	16.60	2.635	±	0.038
7	Vanillic acid	18.60	0.468	±	0.018
8	Caffeic acid	19.00	0.411	±	0.005
9	Epicatechin	19.41	3.392	±	0.119
10	Syringic acid	20.05	0.329	±	0.010
11	Syringaldehyde	21.80	0.441	±	0.004
12	Chicoric acid	22.21	0.486	±	0.002

13	p-Coumaric acid	23.06	70.167	±	0.346
14	t-Ferulic acid	24.00	1.752	±	0.030
15	Benzoic acid	26.38	8.939	±	0.063
16	Hyperoside	26.92	8.931	±	0.077
17	Rutin	27.16	0.716	±	0.135
18	Resveratrol	27.70	2.675	±	0.022
19	Rosmarinic acid	28.53	60.682	±	1.002
20	t-Cinnamic acid	34.39		n.q.	
21	Quercetin	35.89	1.606	±	0.001
22	Hesperetin	39.30	0.338	±	0.060
23	Kaempferol	41.74	1.692	±	0.003

1.2. Western Blot

Caco-2 cells (1*10[6] cells/well) were seeded into 6-well plates and left to adhere overnight. Cells were treated with LPS (10 μg/ml) and/or increasing concentrations of PPE (1-10-100 μg/ml) for 24 and 48 hours. Protein expression of ZO-1 was assessed by Western blot, as previously described [3]. Briefly, cells were lysed, and protein concentrations were determined using the Bradford assay. Equal amounts of protein (30 μg) were loaded and separated by SDS-PAGE, followed by transfer to PVDF membranes. The membranes were blocked for 1 hour in 5% non-fat milk in PBS-Tween20 (0.1%), then incubated overnight at 4°C with a primary antibody against ZO-1 (1:1000, Cell Signaling Technology, Danvers, MA, USA). After washing, membranes were incubated with a secondary antibody (1:5000, Bio-Rad, Milan, Italy) for 1 hour at room temperature.
Membranes were developed using ECL Western Blotting Detection Reagents (Bio-Rad) and analyzed using Alliance 1D software (UVITEC, Cambridge, United Kingdom) and ImageJ software. Band intensity was quantified and normalized to the expression of β-actin to ensure equal protein loading across samples.

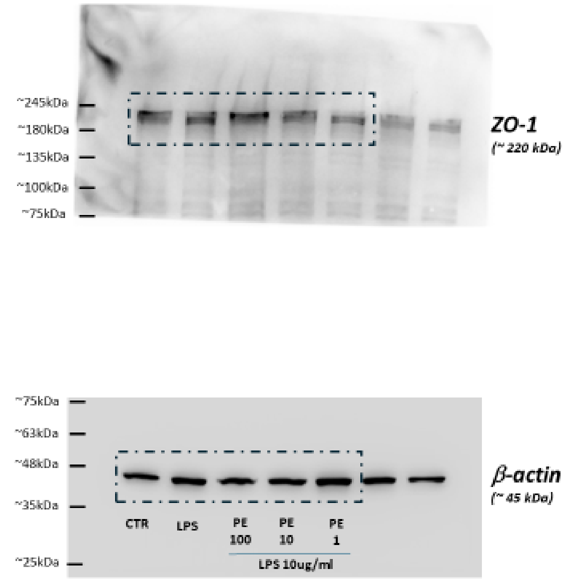

Uncropped of Western Blot reported in Figure 6A

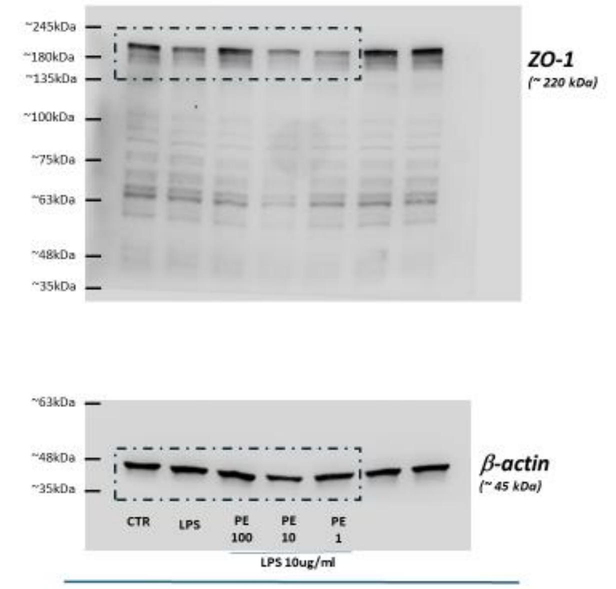

Uncropped of Western Blot reported in Figure 6B

References:

1. Ignat, I., Volf, I., Popa, V.I. A critical review of methods for characterisation of polyphenolic compounds in fruits and vegetables. *Food Chem.* **2011**, *126*, 1821–1835.
2. Di Simone, S.C., Libero, M.L., Pulcini, R., Nilofar, N., Chiavaroli, A., Tunali, F., Angelini, P., Flores, G.A., Venanzoni, R., Cusumano, G., Zengin, G., Brunetti, L., Recinella, L., Leone, S., Orlando, G., Menghini, L., Ferrante, C., Acquaviva, A. Phytochemical profiling and biological evaluation of the residues from industrial hemp (*Cannabis sativa* L.) inflorescences trimming: Focus on water extract. *Food Biosci.* **2024,** *62*, 105344.
3. Gatta, M., Dovizio, M., Milillo, C., Ruggieri, A.G., Sallese, M., Antonucci, I., Trofimov, A., Khavinson, V., Trofimova, S., Bruno, A., Ballerini, P. The Antioxidant Tetrapeptide Epitalon Enhances Delayed Wound Healing in an in Vitro Model of Diabetic Retinopathy. *Stem Cell Rev and Rep*. **2025**, *21*, 1822–1834.
